# Supplementary material for: Modelling Skylarks (Alauda arvensis) to Predict Impacts of Changes in Land Management and Policy: Development and Testing of an Agent-Based Model
Source: PLoS One. 2013 Jun 6;8(6):e65803. doi: 10.1371/journal.pone.0065803 (PMC3675089; doi:10.1371/journal.pone.0065803)
Supplement: Supporting Information S4 — The skylark ODdox as a zipped archive. (ZIP) [file pone.0065803.s004.zip › Skylark_ODdox/pesticide_8h.html]

ALMaSS Skylark ODdox: pesticide.h File Reference


ALMaSS Skylark ODdox
 2.0


- Main Page
- Related Pages
- Classes
- Files

- File List
- File Members

Classes |
Macros |
Variables

pesticide.h File Reference

|  |  |
| --- | --- |
| Classes | |
| struct | Diffusor |
| class | Pesticide |
| struct | PesticideEvent |

|  |  |
| --- | --- |
| Macros | |
| #define | PEST\_DEBUG |
| #define | PEST\_GRIDAREA   16 |
| #define | PEST\_GRIDSIZE   4 |
| #define | PEST\_GRIDSIZE\_POW2   2 |
| #define | PEST\_WATER\_CHECK |

|  |  |
| --- | --- |
| Variables | |
| class Pesticide \* | g\_pest |

---

## Macro Definition Documentation

#define PEST\_DEBUG

#define PEST\_GRIDAREA   16

Referenced by Pesticide::Pesticide(), and Pesticide::TwinMapSprayCorrectBorders().

#define PEST\_GRIDSIZE   4

Referenced by Landscape::ChangeMapMapping(), and Pesticide::Pesticide().

#define PEST\_GRIDSIZE\_POW2   2

Referenced by Landscape::ChangeMapMapping(), Pesticide::Pesticide(), Pesticide::SupplyPesticide(), and Pesticide::TwinMapSprayPixel().

#define PEST\_WATER\_CHECK

---

## Variable Documentation

class Pesticide\* g\_pest


- CJT
- MSVC
- ALMaSS Working Source
- Landscape
- pesticide.h
- Generated on Thu Jan 10 2013 13:15:35 for ALMaSS Skylark ODdox by
   1.8.1.1
